# Relationship between Oxidative Stress, Circadian Rhythms, and AMD

**DOI:** 10.1155/2016/7420637

**Published:** 2015-12-28

**Authors:** María Luisa Fanjul-Moles, Germán Octavio López-Riquelme

**Affiliations:** ^1^Facultad de Ciencias, Universidad Nacional Autónoma de México, Ciudad Universitaria, 04510 Ciudad de México, DF, Mexico; ^2^Posgrado en Ciencias Cognitivas, Universidad Autónoma del Estado de Morelos, Facultad de Humanidades, UAEM, Avenida Universidad No. 1001, Colonia Chamilpa, 62209 Cuernavaca, MOR, Mexico

## Abstract

This work reviews concepts regarding oxidative stress and the mechanisms by which endogenous and exogenous factors produce reactive oxygen species (ROS). It also surveys the relationships between oxidative stress, circadian rhythms, and retinal damage in humans, particularly those related to light and photodamage. In the first section, the production of ROS by different cell organelles and biomolecules and the antioxidant mechanisms that antagonize this damage are reviewed. The second section includes a brief review of circadian clocks and their relationship with the cellular redox state. In the third part of this work, the relationship between retinal damage and ROS is described. The last part of this work focuses on retinal degenerative pathology, age-related macular degeneration, and the relationships between this pathology, ROS, and light. Finally, the possible interactions between the retinal pigment epithelium (RPE), circadian rhythms, and this pathology are discussed.

## 1. Introduction

Over millions of years of evolution, organisms have developed diverse protective systems to control excess reactive oxygen species (ROS), which produce oxidative stress (OS). This term refers to elevated intracellular levels of ROS that cause damage to lipids, proteins, and DNA, a process that has been considered to be linked to a myriad of pathologies in humans.

The mechanisms of ROS production (e.g., via aerobic respiration or flavin-containing oxidases) and its rapid removal (e.g., via catalase) are present in almost all of the cell types found in organisms. OS effects depend on the intensity of damage induced by the ROS in the cell and the cellular response to this damage; if the cell is unable to overcome the damage and recover its function or if exogenous and endogenous antioxidant defenses (AOXs) cannot counter it, the cell can die. However, ROS have also been shown to function as second messengers by transducing extracellular signals to generate specific cellular responses. Proteins and other molecules that participate in signaling pathways can be modified by redox changes [1]. Numerous studies have substantially contributed to the development of the concepts of OS and the mechanisms involved in the production and regulation of ROS as well as their participation in cellular signaling processes (for a review, see [2]). The present work briefly reviews some of the mechanisms of ROS production and scavenging in addition to the participation of ROS in complex processes such as aging and biological rhythms. Due to the growing interest in the involvement of ROS in degenerative pathologies, the last part of this review is focused on the effects of OS on retinal degenerative processes, particularly on age-related macular degeneration (AMD). Due to the importance of circadian rhythms in the development of degenerative pathologies, a short survey of the possible relationships between circadian rhythms and AMD is included in the last part of this work.

## 2. Reactive Oxygen Species

**Figure 1:**
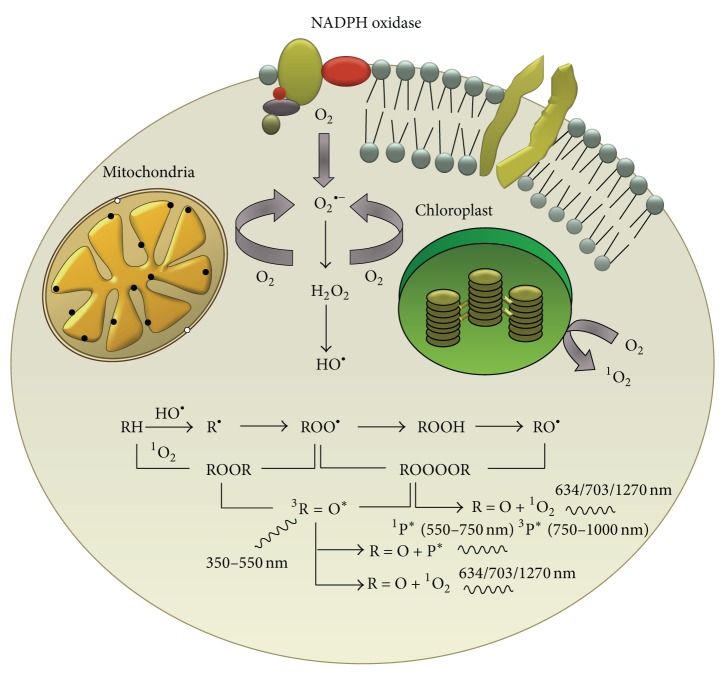
A model showing the formation of reactive oxygen species (ROS) in different organelles of the cell. Superoxide anion radical (O_2_^−^) is produced via the membrane-bound enzyme complex NADPH oxidase (nicotinamide adenine dinucleotide phosphate-oxidase) which is found embedded within the plasma membranes and membranes of various organelles such as mitochondria, chloroplasts, and phagosomes. The dismutation of O_2_^−^ is accompanied by the formation of hydrogen peroxide (H_2_O_2_) and then the hydroxyl radical (HO^•^) via the Fenton reaction. The highly reactive HO^•^ has the capability to oxidize all types of biomolecules such as lipids, proteins, and nucleic acids. The oxidation of biomolecules is accompanied by the formation of high-energy intermediates such as dioxetane (ROOR) and tetroxide (ROOOOR), which upon further decomposition, generate electronically excited species such as triplet excited carbonyl, singlet and triplet excited pigments, and singlet oxygen (^1^O_2_). From Pospišíl et al. [7] with the permission of the authors and Elsevier.

ROS are formed either during metabolic processes that are linked to life-sustaining, enzyme-catalyzed reactions, such as aerobic respiration, or during responses to stress reactions when organisms, including humans, are exposed to biotic and abiotic stress factors, such as situations like hypoxia or anoxia. ROS encompass a variety of diverse chemical species including singlet oxygen, superoxide anion radical, hydroxyl radical (OH), hydrogen peroxide (H_2_O_2_), hydroxylperoxyl radical, alkoxyl radicals, and peroxyl radicals. Superoxide anion (O^−^) is converted to H_2_O_2_ by the enzyme superoxide dismutase (SOD), and the hydroxyl radical (OH) is a byproduct of the Fenton reaction. Nitric oxide (NO) and singlet oxygen are examples of reactive species. Some of these species, such as superoxide or hydroxyl radicals, are extremely unstable, whereas others, such as H_2_O_2_, are freely diffusible and relatively long-lived. These various radical species can be generated either exogenously by physical or chemical factors that induce stress reactions or through cell-dependent mechanisms via several different mechanisms, such as cytosolic enzyme systems or mitochondrial mechanisms. The cytosolic systems include, among others, the family of NADPH oxidases (NOX) [3], whereas the production of mitochondrial superoxide radicals occurs primarily at two discrete points in the electron transport chain (ETC), namely, complex I (NADH dehydrogenase) and complex III (ubiquinone-cytochrome c reductase) [4]. Mitochondrial production of ROS will be discussed in the next section. Some of the exogenous chemical sources of ROS include the xanthine/xanthine oxidase system, which produces O^−^; the Fenton reagent, which generates HO^−^; and photosensitizers such as rose bengal and benzoporphyrin derivatives, which produce ^1^O_2_ upon photosensitization [5, 6]. Physical abiotic factors such as UV radiation and visible light result in the formation of radical (O^−^ and H^•^) and nonradical (^1^O_2_) ROS by Type I and Type II reactions, respectively (for a review, see [7]) (Figure 1). Various endogenous pigments in organisms, such as porphyrins, bilirubins, melanins, and pterins, are known to act as photosensitizers by absorbing radiation or visible light. This leads to the formation of the singlet photosensitizer state, which forms the triplet excited state. The excited photosensitizer undergoes either electron transport, forming O_2_^−^, H_2_O_2_, and HO^•^ or energy transfer, forming ^1^O_2_ (Type II reaction) [8, 9]. In Type I reaction, electron transport leads to the production of O_2_^−^ via the formation of photosensitizer anion radicals and substrate cation radicals or* vice versa* [10]. Spontaneous or enzymatically driven dismutation of O_2_^−^ leads to the formation of H_2_O_2_, which subsequently forms OH^•^ via the Fenton reaction or other metal-catalyzed reactions. In a Type II reaction, triplet-singlet energy transfer from the excited photosensitizer to molecular oxygen forms ^1^O_2_ [11].

Among all of the biomolecules that can be attacked by ROS, lipids are likely the most susceptible to oxidation. All cell membranes are rich in polyunsaturated fatty acids (PUFAs), which are easily injured by oxidizing agents. Peroxidation of cellular lipids modifies membrane properties and produces cytotoxic compounds, although some peroxidation products also play useful roles. Lipid peroxidation (autoxidation), a process that leads to the oxidation of PUFAs due to the presence of several double bonds in their structure, involves the production of peroxides and reactive organic free radicals (FRs). The latter can then react with other fatty acids, initiating a FR reaction cascade.

ROS can also react with nucleic acids by attacking nitrogenous bases and the sugar phosphate backbone. Furthermore, some of their primary effects include basic DNA damage and single- and double-strand DNA breaks. To a large extent, the oxidative radical damage that occurs in nucleic acids results from the reaction of DNA with radicals such as H^•^ and ^•^OH. Hydroxyl radicals are known to attack DNA, causing single- and/or double-strand breaks as well as pyrimidine and purine lesions, both of which can affect the integrity of the genome [12]. The inability of cells to repair the damage incurred in this process may lead to their death; alternatively, mutations may occur in the DNA, leading to carcinogenesis or the development of neurodegenerative diseases [13].

Mitochondrial DNA (mtDNA) is more susceptible to oxidative damage than its nuclear counterpart. Mutations in mtDNA can cause disturbances in the respiratory chain and loss of the control of ROS production. mtDNA is not protected by histones or other associated proteins, and because it has intronless regions and a high transcription rate, mtDNA is more susceptible to oxidative modifications in its coding regions. The reduced effectiveness of the repair system for mtDNA damage may be the cause of the accumulation of OS and its consequences. The formation of carbonyl derivatives is the most widely studied OS-induced protein modification [14]. Carbonyl formation can occur through a variety of mechanisms, including the direct oxidation of certain amino acid side chains and oxidation-induced peptide cleavage. Although all organs and proteins can potentially be modified by OS, certain tissues and protein targets may be especially susceptible [3]. Other protein modifications are NO-dependent. For example, NO reacts with O_2_^•−^ to generate ONOO^−^, which is capable of initiating further protein oxidation and nitration [15, 16].

The nitrogen dioxide radical, which is biologically formed from the reaction of N_2_ with oxygen or by the decomposition of ONOO^−^, reacts with tyrosine residues, resulting in the formation of 3-nitro-tyrosine. The addition of NO to the thiol groups of proteins via S-nitrosation (also referred to as S-nitrosylation) has also been reported to be associated with neurodegenerative diseases [16].

### 2.1. Mitochondria and ROS

Mitochondria are a major intracellular generator of ROS (mitoROS) [17]. Multiple producers of O^−^ have been reported in mitochondria, including flavins in complexes I and II; ubiquinone binding sites in complexes I, II, and III; flavoprotein-Q oxidoreductase-mediated fatty acid beta-oxidation; and glycerol-3-phosphate, 2-oxoglutarate, and pyruvate dehydrogenases [18–21]. The major players in mitoROS production are complexes I and III of the mitochondrial ETC [18], although other ROS-producing mitochondrial sites include mitochondrial glycerol-3-phosphate dehydrogenase, the electron-transferring flavoprotein/ETF: ubiquinone oxidoreductase system of fatty acid oxidation, dihydroorotate dehydrogenase, the dihydrolipoamide dehydrogenase, and 2-oxoacid dehydrogenase complexes. In addition, the 2-oxoglutarate dehydrogenase complex, the branched-chain 2-oxoacid dehydrogenase complex, the pyruvate dehydrogenase complex, and proline dehydrogenase, among others, have been implicated in ROS production (for a review, see [22]). The mitochondrial ETC consists of four mitochondrial redox carriers that are known as complexes I, II, III, and IV. Electrons that are donated by NADH and FADH_2_ toward complexes I and II, respectively, are transferred to complex III and then eventually to complex IV to reduce oxygen molecules to water [23]. In complex I, O^−^ production is driven by the presence of NADH, which donates electrons to a series of redox enzymes within the complex itself; this series includes flavin mononucleotide, Fe^−^S clusters, and coenzyme Q10 (ubiquinone) [24]. O^−^ production has been linked to two sites within complex I: reduced flavin mononucleotides and quinone binding sites [24]. In isolated mitochondria, complex I can reduce O_2_ to O^−^ under two different conditions: (1) when ATP is in low demand and the proton motive force (PMF) is maximal and (2) when the NADH/NAD+ ratio is high and ATP is continuously synthesized, resulting in a low PMF [25]. The first mechanism, which is also known as reverse electron transfer (RET), occurs when the PMF is maximal or when electrons donated from succinates in complex II are reverse transferred to complex I. This action causes electron binding to quinone binding sites, which allows electrons to leak out and reduce oxygen molecules [25]. The second mechanism, which is known as forward electron transfer, requires the donation of electrons from reduced form to oxygen molecules, producing O^−^. This process depends on a high NADH/NAD+ ratio or inhibition of the respiratory chain through mitochondrial damage, ischemia, the loss of cytochrome c, or mutations. It also causes the loading of electrons onto flavin and the subsequent leakage of electrons that reduce oxygen molecules.

In addition to the NADH-driven ROS production from complex I and, subsequently, complex III, complex II has also been demonstrated to be a source of ROS production [26, 27]. Complex II, or succinate dehydrogenase, is composed of four subunits: flavoprotein, the Fe–S cluster, and two transmembrane cytochrome b heme subunits [21, 28]. In complex II, O^−^ production is driven by the presence of succinate, which sequentially donates two electrons to flavin, the Fe–S cluster and ubiquinone [27, 29, 30]. Complex IV, in contrast, has yet to be shown to directly produce ROS, although it affects the course of O^−^ production by complexes I, II, and III and seems to be the terminal and rate-limiting complex that influences the flow of electrons across the ETC.

### 2.2. Autophagy and ROS

Autophagy is a lysosome-mediated degradation process for nonessential or damaged cellular components [31]. Physiologically, autophagy preserves the balance between organelle biogenesis and both the synthesis and clearance of proteins. This process is emerging as an important mediator of pathological responses because it involves cross talk between ROS and RNS (reactive nitrogen species) during both cell signaling and protein damage. Dysregulated redox signaling or mitochondrial dysfunction can also influence autophagic activities. OS is inseparably linked to mitochondrial dysfunction because mitochondria are both generators and targets of reactive species. Mitochondrial turnover depends on autophagy, which declines with age and is frequently dysfunctional in neurodegenerative diseases [32]. OS can also lead to nonspecific posttranslational modification of proteins, and it contributes to protein aggregation (for a review, see [33]).

### 2.3. Antioxidants 

Organisms have natural AOXs that diminish the harmful effects of continuous ROS production. There are both endogenous and exogenous defense mechanisms against oxidative attack. OS and its biomarkers are the biochemical products of an imbalance between ROS production and the ability of biological AOXs to counteract the effects of ROS metabolites [34]. These defenses are located in the cytoplasm, the cellular membrane, and the extracellular space, and they include enzymatic defense systems, FR scavengers, and chelating agents for transitional metals. The enzymatic systems consist of intracellular molecules, such as SODs, catalase, glutathione peroxidase (GPx), and reductase [35]. FR scavengers slow down oxidation reactions, trapping FRs and transforming them into less aggressive compounds. They can be hydrosoluble and cytosolic (e.g., GSH and vitamin C) or liposoluble and membrane-bound (e.g., vitamin E and carotenoids). Vitamin C is considered to be the main hydrosoluble AOX system, and it may also regenerate tocopherol (vitamin E), which is one of the main liposoluble AOXs in cellular membranes, along with carotenoids (*β*-carotene, lutein, zeaxanthin, and lycopenes). GSH is considered the most abundant AOX system in the cytosol, nucleus, and mitochondria. The relevant chelating agents include molecules that bind to iron and copper as well as flavonoids. In this way, these chelating agents prevent these metals from participating in Fenton and Haber-Weiss reactions. Depending on their source, AOX agents can be either endogenous, as in GSH, SOD, and CAT, or exogenous (only obtained from nutrients), as in vitamins C and E, carotenoids, flavonoids, and oligo elements [36].

#### 2.3.1. Melatonin as an Antioxidant

Melatonin (*N*-acetyl-5-methoxytryptamine) is an important AOX molecule. It is a ubiquitous substance that is secreted by the pineal gland of all mammals, including humans. In addition, its presence has been confirmed in many animals, plants, and unicellular organisms [37–39]. Melatonin participates in diverse functions in the body, including sleep, circadian rhythm regulation, and immunoregulation, and it may have anticarcinogenic effects [39]. As a chelating agent, melatonin is a potent FR scavenger and a regulator of redox-active enzymes [40]. This hormone is secreted during darkness and plays a key role in various physiological responses, including the regulation of circadian rhythms, sleep, homeostasis, retinal neuromodulation, and vasomotor responses. It scavenges hydroxyl, carbonate, and various organic radicals and a number of RNS. Melatonin also enhances the AOX potential of cells by stimulating the synthesis of AOX enzymes, including SOD, GPx, and glutathione reductase, and by augmenting glutathione levels [41]. This hormone preserves mitochondrial homeostasis, reduces FR generation, and protects mitochondrial ATP synthesis by stimulating complexes I and IV, thereby counteracting oxidative mtDNA damage and restoring the mitochondrial respiratory control system [41]. Because reduced complex I activity and is a sign of enhanced electron leakage, the resulting increase in OS is sufficient to induce apoptosis. The ability of melatonin to return complex I activity to normal levels suggests its significance in overall health via the prevention of age-associated degenerative changes [42].

### 2.4. ROS and Aging

Aging has been considered to involve the progressive accumulation of changes with time that are associated with or responsible for the ever-increasing susceptibility to disease and death that accompanies advancing age. In 1956, Harman [43] proposed that endogenously generated oxygen FRs induce the macromolecular oxidative damage that is responsible for senescence (for a review, see [44]) and is associated with a decline in physiological fitness during aging. This “free radical hypothesis” was modified and merged with the “oxidative stress hypothesis,” resulting in the term “oxidative stress” [45], which was defined as a disturbance in the pro-oxidant-antioxidant balance that results in a cellular state in which the AOXs are insufficient for complete eradication of various ROS [44]. Both propositions postulate that the progression of age-related deleterious alterations is a function of the imbalance between ROS fluxes and AOXs, and both predict that the narrowing of this gap should reduce the amount of structural damage and thereby prolong the life span. “Therefore, from this historical perspective, the postulated mechanism by which ROS are implicated in the aging process can be aptly characterized as the structural damage-based oxidative stress hypothesis” [44]. Over the past several decades, however, there has been a shift in ideology concerning the role of ROS in cell physiology. Some oxidants, particularly H_2_O_2_, have now been recognized as essential for cell survival due to their regulatory roles in a wide range of functions, including gene regulation, cell signaling, protein activation/deactivation, cellular differentiation, and apoptosis (reviewed in [46, 47]). Now, ROS have been recognized to act as signals [48, 49]. Although some of these signaling pathways promote cell death [50], others, such as oxygen-sensing and hypoxic responses [51] or the induction of autophagy [52], promote cell survival. Therefore, in theory, low (nontoxic) levels of ROS can promote lifespan extension by activating pathways that promote cellular resistance to various stresses.

Although there is little doubt that high levels of ROS are detrimental, mounting evidence indicates that mild to moderate ROS elevation extends lifespan. Dietary caloric restriction, inhibition of insulin-like growth factor-I signaling, and inhibition of the nutrient-sensing mechanistic target of rapamycin are robust longevity-promoting interventions [53] that all appear to elicit retrograde mitochondrial signaling processes that may even spread to other cells.

Other factors, such as biological rhythms, also seem to contribute to aging [54]. Age-associated changes in the day/night rhythm of melatonin production have been identified, with phase advances encountered more frequently in the elderly compared with young women [55]. Suprachiasmatic nucleus (SCN) function has also been shown to decline with age, particularly in patients with aging-associated neurodegenerative disorders, which are a major cause of dementia, and other poor health conditions that are common in elderly populations [56, 57]. A decline in melatonin production and altered melatonin rhythms can be major contributing factors to increased levels of OS and the associated degenerative changes that are observed in the elderly. Nevertheless, individuals of the same chronological age can exhibit dissimilar degrees of senescence-associated functional impairment, differences that may be attributable to well-documented interindividual variations in melatonin levels [58–61]. Variations in the degenerative changes that occur in cells and tissues have been attributed to variations in melatonin production; these changes are more often determined by an individual's physiological age rather than his chronological age [62]. Recently, genetic variation in the enzyme ASMT (HIOMT), which performs a metabolic step that determines the amount of melatonin produced [63], has been demonstrated.

## 3. Circadian Clocks and ROS

Circadian rhythmicity is a fundamental biological phenomenon that is universally important. This endogenous, innate oscillation with a period of approximately one day has been identified in all of the organisms that have been studied to date, from bacteria to eukaryotes. Temporal variations that are driven by a circadian oscillator are evident in many cellular functions including gene expression, metabolic flux rates, concentrations of signaling molecule, and cellular substructures. In multicellular organisms, circadian rhythms can be studied at different integration levels, from cell-to-cell interactions to organ physiology and from endocrine and neural communications to behavior. Although the control and coordination of circadian rhythms in metazoans are typically organized by specialized pacemaker structures, primary oscillations are generated at the cellular level. These rhythms are widely accepted to be genetically determined, and several genetic clocks have been identified in different taxa, including unicellular organisms [64].

The core of the circadian clock is based on an intracellular time-tracking system that enables organisms to anticipate environmental changes and thereby adapt their behavior and physiology to the appropriate time of day [65]. It is well known that in some animals, such as insects and mammals, a specific set of transcription factors constitutes the molecular architecture of the circadian clock. These factors are organized into positive and negative regulatory feedback loops that function in a cell-autonomous manner and that are rhythmically controlled by a master oscillatory system, which coordinates tissue-specific rhythms according to the input it receives from the rhythms of the outside world [66].

The one or more endogenous oscillators that function to generate a free-running period that is close to 24 h when the organism is maintained under constant environmental conditions are a central component of the circadian system. At the molecular level, these oscillators are based both on the products of “clock genes,” which are organized into transcriptional-translational feedback loops (TTLs), and on oscillations in posttranslational modifications of proteins, which contribute significantly to circadian oscillations [67]. Some of the clock genes encode transcriptional activators, whereas others encode negative feedback elements that inhibit their own expression by disrupting the activity of their activators. Meanwhile, kinases and phosphatases regulate the speed and precision of the clock [68]. Components of the oscillators receive environmental information through input pathways, allowing the oscillators to remain synchronized with the 24 h solar day. This time-of-day information from the oscillator(s) is then relayed through output pathways to regulate the expression of circadian clock-controlled genes and overt rhythmicity. One mechanism by which the output pathways are predicted to be rhythmically controlled is through transcription factors or signaling molecules that are themselves components of the oscillator. These factors, which are activated by the circadian clock, may in turn regulate the downstream clock control genes in a time-of-day-specific manner [69].

The internal clock would be useless if it was not able to synchronize with environmental time or if the cells within a tissue were not synchronized to each other. Therefore, input pathways to the circadian oscillator are vital to maintain the proper timing of the oscillator with respect to the environment. In a process called entrainment, input pathways reset the oscillator so that the period of the oscillator conforms to the 24 h period of the environment [69]. Input pathways detect environmental cues and utilize various mechanisms to increase or decrease the levels or activity of components of the molecular oscillator to set the clock to the correct time of day. One of the most ubiquitous time-giving cues is light, but nonphotic environmental cues, including nutrition, temperature, and social interactions, can also entrain the circadian clock [70–73]. In addition, the clock utilizes a strategy called gating to restrict responses to environmental cues at certain times of day. For example, diurnal mammals are typically insensitive to a light pulse during the day, but, during the night, a light pulse can advance or delay the clock to synchronize it with the environment [74], just as we would adjust our watches to match the local time. In organisms of various complexities, cells vary in their ability to support a molecular oscillator that can be entrained by environmental signals. In unicellular organisms, each cell has a fully entrainable oscillator that primarily responds to light [75]. However, in complex multicellular organisms, not all cell types have the necessary sensory capabilities, such as photoreception, to entrain the circadian oscillator. The cellular oscillators and overall rhythmicity of the organism are broken down into components that include a master pacemaker and peripheral oscillators [76]. To integrate multiple sensory inputs, organisms that possess a nervous system typically delegate the ability to sense environmental cues to a central oscillator or pacemaker rather than to individual cells. In mammals, sensory inputs to the clock are integrated in the brain, where signals from the master pacemaker entrain the oscillators in other tissues throughout the organism. Light is perceived by nonvisual retinal ganglion cells that transmit information via neural connections to the master pacemaker, which is located in a region of the hypothalamus, the SCN. The SCN pacemaker synchronizes oscillators in other tissues by a mechanism that utilizes circadian input pathways from the SCN to individual cells in the periphery. In addition to maintaining the entrainment of peripheral oscillators by the environment, this system ensures that cellular oscillations within tissues are properly in phase to provide resonance between individual cellular rhythms [77].

Melatonin acts as an important synchronizer in mammals and provides temporal feedback to oscillators within the SCN, which regulates the circadian phase and maintains rhythmic stability [78]. Various studies in birds and mammals, both in vitro and in vivo, have demonstrated that melatonin adjusts the circadian “clock” by acting directly on the molecular timing system [78, 79]. In humans, as in other mammals, melatonin is presumed to influence circadian rhythms by acting directly on receptors in the SCN [80].

For at least three decades, the cellular redox state in plants and animals has been known to change over circadian time [81], although many chronobiologists have long assumed that metabolic rhythms are a functional readout of the circadian clock and that redox oscillators simply provide feedback to the central TTL pacemaker [82].

Recent discoveries have, however, uncovered redox-based circadian oscillators that are conserved across both eukaryotic and prokaryotic species. Circadian rhythms in ROS generation and scavenging have been observed in a variety of species, including fungi [83], plants [84], and animals [85]. Further evidence of the involvement of the circadian clockwork in the regulation of redox systems is supported by studies of mutants, where the deletion or disruption of clock genes also results in the perturbation of redox systems. In mammals, 24 h oscillations in concentrations of oxidized NADPH and the reduced form of FAD have been observed in organotypic slices of the rodent SCN [86]. The rhythms of these coenzymes are believed to be dependent on the molecular clockwork because Bmal1^−/−^ mice exhibit stochastic, but not circadian, FAD, and NADPH rhythms. These studies also revealed links between the redox state and the membrane excitability of SCN neurons, given that oxidizing and reducing agents can produce hyperpolarization and depolarization, respectively. Redox-dependent modulation of K^+^ channel conductance is believed to underlie these oscillations [86]. All of these findings support complex interdependence among the redox state, cellular energetics, and circadian clockwork in mammals.

The links between circadian physiology, prooxidizing changes in the redox state, as reflected by a decline in redox potential, and the process of aging seem to be coherent and well established [87]. Hence, in the present work, the OS damage generated in the human eye and its relationship with AMD is briefly discussed. Here, the impact of OS from the accumulation of ROS, which is probably due to circadian alterations, and its relevance to chronic pathologies, is briefly reviewed, with a particular emphasis on retinal neurodegenerative diseases such as AMD.

## 4. The Retina and Oxidative Stress

In humans, the retina is very prone to the generation of ROS compared with other tissues. This structure is a photosensitive tissue with high oxygen levels in the choroid and a high metabolic rate that is also exposed to light. Furthermore, the retina contains a higher concentration of PUFAs than other body tissues [88]. Lipids in the outer segment membranes of photoreceptors can be oxidized by radicals produced during photonic activation, and the endogenous oxygen species that are generated in the eyes through this process can induce ROS-related acute or chronic retinal damage. All of these factors, combined with the very high oxygen levels in the choroid, the high metabolic rate, and the exposure to light, make our retinas vulnerable to the effects of light, especially to light of shorter wavelengths [89]. “Each day, the retina of the average human absorbs approximately 10^12^ to 10^15^ photons, and this amount can be greatly increased by the workplace, sunlight exposure, or medical imaging of the retina during an eye examination. Such high levels of exposure to visible light can cause irreparable damage to the retina” [90]. Recently, Roehlecke et al. [91] demonstrated that ROS are generated and OS occurs directly in the outer segments of photoreceptors after blue light irradiation.

Among all of the retinal cell organelles, the mitochondria are particularly sensitive to OS due to their handling of electrons in the respiratory chain. In addition, after blue light exposure, more electrons deviate from the respiratory chain in the mitochondria, resulting in further damage. In fact, inhibiting the mitochondrial transport chain in retinal pigment epithelium (RPE) cells or adding mitochondria-specific AOXs blocks ROS formation and cell death [89]. Furthermore, chromophores in general and cytochromes in particular can be sources of ROS [36]. OS-induced inflammation initiates a functional decline in tear production, and dry eye is the first symptom of this type of damage to the eye [92].

In the human eye, OS in the RPE is widely accepted as a contributing factor for retinal disorders. The RPE, a monostratified cell layer, constitutes the outer blood-retinal barrier (BRB), controls fluid and metabolic exchange between the retina and the choriocapillaris, and participates in several functions linked to photoreceptor physiology, such as the phagocytosis of shed photoreceptor outer segments and transportation of molecules to and from the retina [93].

In addition, RPE cells release factors that control neuronal survival and angiogenesis (e.g., pigment epithelium-derived factor, PEDF) and that promote photoreceptor survival and have an antiangiogenic effect on the choriocapillaris [94, 95]. The RPE secretes PEDF on the apical side; in contrast, the angiogenic factor vascular endothelial growth factor (VEGF) is secreted on the basolateral side to maintain fenestration of the choriocapillaris. An imbalance in the expression of PEDF and VEGF appears to be involved in ocular pathologies. Because PEDF has antioxidative and antiangiogenic properties, this factor may protect against vision-threatening angiogenic mechanisms. In contrast, VEGF is generally accepted as the most potent inducer of endothelial activation and angiogenesis, a process in which new vessels develop from the preexisting vasculature (for a review, see [96]). Under ischemic or hypoxic conditions, which occur in many neovascular diseases, retinal expression and production of VEGF are dramatically increased [97, 98].

The oxidation of polyunsaturated fats in the retina leads to lipid peroxidation products such as carboxyethylpyrrole and 4-hydroxy-2-nonenal, which can form adducts with proteins and accumulate in the outer retina and in drusen [99]. The levels of AOX proteins such as catalase and SOD are increased in RPE homogenates derived from the eyes of late-stage AMD patients, indicating a response to increased OS in the eyes of these patients [100].

As indicated in previous sections, the NOX [101] family of enzymes has recently been recognized as a generator of ROS in rod or cone photoreceptors after they are damaged due to serum deprivation in a model of retinitis pigmentosa [102]. Roehlecke et al. [103] further demonstrated that blue light irradiation results in increased superoxide anion production.

Lipofuscin is the generic name given to a heterogeneous group of complex and autofluorescent bisretinoids, lipid peroxides, and proteins and to various fluorescent compounds that are formed from modified lipids or that are derived from vitamin A. The major substrate for lipofuscin in the RPE is a nondegradable end product that results from phagocytosis of the photoreceptor outer segments, which are rich in PUFAs and vitamin A. This substrate is located within the RPE, where it accumulates in lysosomes with age. Lipofuscin is a byproduct of the visual cycle [104, 105] that is produced when phagocytosed material is not entirely degraded within the RPE lysosomes, resulting in the accumulation of this complex over time. Lipofuscin is continually exposed to high oxygen tensions (70 mmHg) and to visible light (400–700 nm) during daylight hours, creating a prime environment for the generation of ROS that have the potential to damage cellular proteins and lipid membranes. Thus, lipofuscin is a photoinducible generator of ROS that increases the risk of oxidative injury [106]. A lipofuscin fluorophore, A2E, is now known to mediate the blue light-induced apoptosis of RPE cells, which inhibits the degradative capacity of lysosomes and disrupts membrane integrity [107, 108]. This complex phototoxic effect is wavelength-dependent; more superoxide anions are generated in granules exposed to blue light (400–520 nm) than in granules exposed to red light (660–730 nm) or full white light [109]. Lipofuscin induces the apoptosis of cultured RPE cells, leading to a decline in mitochondrial activity that is associated with the translocation of cytochrome c, an apoptosis-inducing factor, and two apoptosis-inducing proteins into the cytoplasm and nucleus [110].

However, whether A2E or lipofuscin may impair lysosomal function is still controversial. Recently, Saadat et al. [111] demonstrated that A2E and impaired autophagy mediate RPE cell damage. These authors proposed that decreased lysosomal capacity might also result in decreased autophagy in RPE cells, which would not affect autophagosome biogenesis or fusion with lysosomes but would instead impair the terminal stage of the degradation process. Thus, the accumulation of A2E in aged RPE cells, in which autophagy is already impaired, may result in RPE cell damage. The authors also considered the possibility that A2E inhibited lysosomal function, which then resulted in the accumulation of autophagosomes rather than an increase in autophagic flux through this pathway.

Some authors have maintained [112] that the RPE secretes apolipoprotein B particles into Bruch's membrane and that these particles accumulate with age and may in turn form a lipid wall, a precursor of the basal linear deposit. Then, certain constituents of the described aggregates may interact with ROS, resulting in proinflammatory, peroxidized lipids and leading to the upregulation of cytokines/chemokines, which then promotes neovascularization. However, recent publications indicate that, in the RPE, OS is also capable of inducing protective pathways, such as the phosphatidylinositide 3-kinase (PI3K)/Akt and nuclear factor erythroid-2-related factor 2 pathways. In addition, VEGF and neuroprotectin D1 signaling act to protect the retina [113, 114].

The phagocytosis of oxidized fatty acids from photoreceptor outer segments contributes to metabolic failure in the RPE [115]. Importantly, the FR byproducts of mitochondrial energy metabolism damage the RPE [116]. In addition, the mitochondrial ETC generates superoxide radicals through single-electron leaks at respiratory complexes I and III [18].

Flavin-dependent enzymes in the mitochondrial matrix may also be large contributors of ROS [22]. Superoxide can directly damage mitochondrial DNA, proteins, and lipids, and it can also be converted to H_2_O_2_ by manganese SOD in the mitochondria [22]. The potential for RPE light damage is influenced by a number of factors, including age, diurnal fluctuations, and pathology [117].

Otherwise, circadian photoreception decreases with age due to disruptions to circadian rhythms, age-related pupillary miosis and reduced crystalline lens transmission, particularly of blue light. Circadian studies have revealed loss of the control of pupil size and the crystalline lens in aging subjects [118].

## 5. ROS and Age-Related Macular Degeneration (AMD)

**Figure 2:**
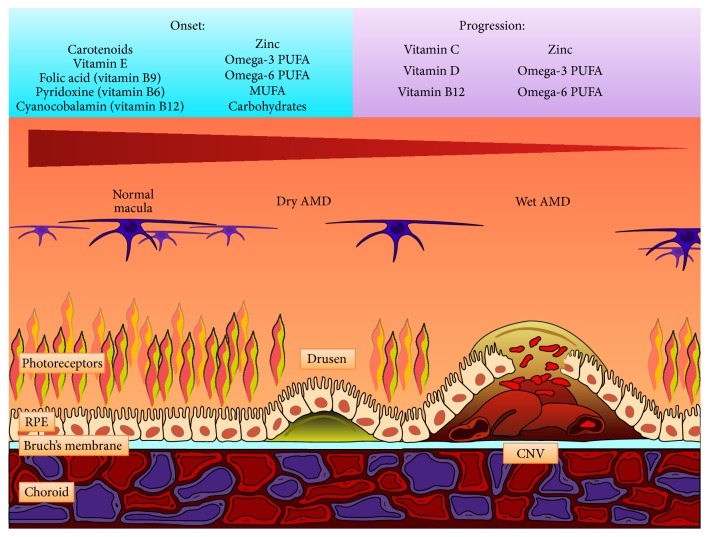
Development of AMD. AMD is mainly due to photochemical damage and oxidative stress. In the figure, the progression of AMD is represented by the cellular structure of the normal macula (left side), the dry AMD (center), and the wet AMD (right side). In the left side of the image, the cells in the macula are normal. In the dry form of AMD (center), drusen impair the metabolic connection between the choroid and the upper layers of macula, leading to the degeneration of the RPE and photoreceptors. In the wet form of AMD (right side), the production of neovascular factors results in the formation of choroidal neovascularization (CNV) with subsequent fluid leakage and major degeneration of the RPE and photoreceptors. In the normal ageing process, lipids accumulate in Bruch's membrane, causing the membrane to thicken and improving the oxidative distress. Moreover, Bruch's membrane lacks an adequate intrinsic antioxidant system. Furthermore, the lipids can bind the macrophages, inducing the secretion of vascular endothelial growth factor (VEGF). The production of inflammatory factors improves the damage to the RPE and photoreceptors and induces the formation of functional microvascular networks (choroidal neovascularization). Antioxidant factors might prevent the disease and delay its progression. In the upper portion of the image, the pool of nutrients involved in the onset (left side) and progression (right side) of AMD are listed. From Zampatti et al. [131], with the permission of the authors and Elsevier. Rightlinks License number is 3679500345328.

AMD, a major cause of visual impairment in the elderly, is linked to pathological changes involving the RPE. AMD is a progressive neurodegenerative disease of the central retinal area (macula lutea) and represents the most common cause of legal blindness in industrialized countries [119]. Epidemiologic studies from several countries have shown a dramatic increase in the prevalence and severity of AMD with age. Despite intensive basic and clinical research, its pathogenesis remains unclear, likely due to its multifactorial nature [120]. In addition to the strong age dependence of the disease, complex interactions between metabolic, functional, genetic, and environmental factors create a platform for the development of chronic changes in the ocular structures of the macular region (e.g., choriocapillaris, Bruch's membrane, RPE, and photoreceptors), and changes in each of these structures may contribute to varying degrees to the onset of AMD (Figure 2). Photoreceptors renew their light-sensitive outer segments continuously and shed their distal tips daily. The adjacent RPE efficiently consumes shed photoreceptor outer segment fragments (POSs) and recycles or digests their components via phagocytosis [121]. Outer segment renewal is crucial for the photoreceptor function and survival. Experimental studies have demonstrated that a lack of efficient POS phagocytosis by RPE cells in some rat strains causes rapid photoreceptor degeneration [122]. A delay in POS digestion can directly cause the accumulation of undigested POS materials, such as lipofuscin, in the RPE, which is detrimental to the RPE and the retina and may contribute to the development or progression of AMD [123, 124].

Based on its clinical presentation, AMD is categorized into early, intermediate, and late stages [125]. Early and intermediate AMD are characterized by soft, yellowish deposits that vary in size from small to large (drusen) and by pigmentation changes in the macula, with little or no visual loss. In late AMD, visual loss appears in two forms, that is, neovascular AMD (also called “wet” or “exudative” AMD), in addition to drusen and atrophy. This form of AMD is characterized by the presence of edema and hemorrhage within or below the retina or RPE and geographic atrophy (also called “dry” AMD). Currently, there are no treatments for geographic atrophy, but neovascular AMD is treated with VEGF inhibitors, which, although not curative, are often effective in preventing severe visual loss [126]. The dry form, also known as age-related maculopathy, is characterized by the presence of drusen under the RPE that is accompanied by either the loss or focal accumulation of melanin pigment. This form of AMD is typically characterized by a progressive course that leads to degeneration of the RPE and photoreceptors. The exudative form is linked to choroidal neovascularization that is directed toward the subretinal macular region, with subsequent bleeding and/or fluid leakage that can result in a sudden loss of central vision. It is the most rapidly progressing form of AMD. Both the atrophic and exudative forms are associated with severe visual impairment [119]. The pathophysiology of AMD is complex, and, in addition to genetic predispositions, at least 4 processes contribute to the disease: lipofuscinogenesis, drusogenesis, local inflammation and neovascularization (in the case of the wet form), and immunological mechanisms [127].

The current pathophysiological conception of AMD assigns a primary role to age-related, cumulative oxidative damage to the RPE due to an imbalance between the generation and elimination of ROS [128]. In particular, lipofuscin has been hypothesized to be the primary source of the ROS responsible for both the cellular and extracellular matrix alterations found in AMD [129, 130].

The accumulation of lipofuscin and other lipid peroxides and potentially toxic substances may dramatically influence RPE physiology, as described above. This accumulation greatly reduces the phagocytic capacity, lysosomal enzyme activities, and AOX potential of the human RPE in vitro [133, 134]. The first clinical sign of early AMD is drusen, as mentioned above. Drusen are lipid-rich, sub-RPE deposits that contain a variety of proteins, including vitronectin, components of the terminal complement cascade, and b-amyloid [135, 136]. Microscopic analysis of eyes donated by patients with AMD revealed lipid deposits within Bruch's membrane and apoptosis of RPE cells as features of the disease that are distinct from normal aging. The advanced form of dry AMD, which leads to geographic atrophy, was characterized by breakdown of the RPE, the choriocapillaris, and photoreceptors in regions of the retina, often where large drusen were present [137]. Dysregulation of the phagocytosis of oxidized fatty acids from photoreceptor outer segments is a contributing factor to the metabolic failure of RPE cells [115, 123]. In addition to all of these factors, the damage caused by the FR byproducts of mitochondrial energy metabolism has been implicated in age-related damage to the RPE [116].

As reviewed above, the mitochondrial ETC generates superoxide radicals through single-electron leaks at respiratory complexes I and III [18], and flavin-dependent enzymes in the mitochondrial matrix may be large contributors of ROS [29].

**Figure 3:**
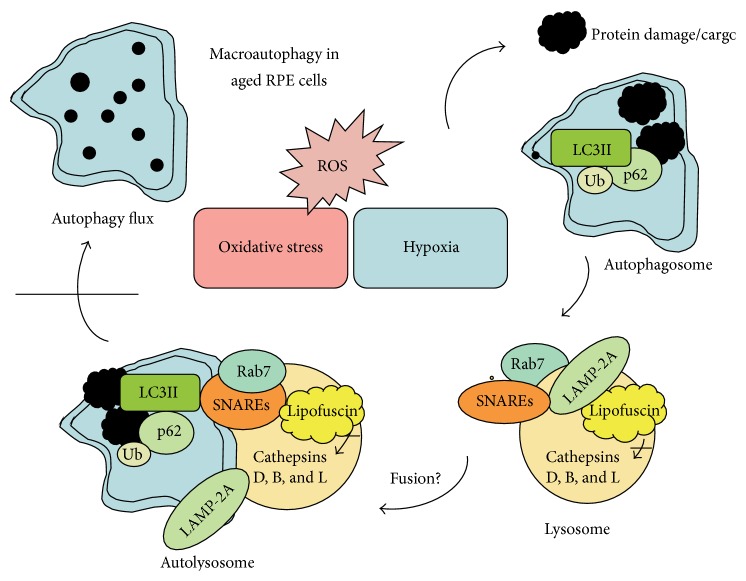
Schematic presentation of the macroautophagy process in aged retinal pigment epithelial (RPE) cells. Oxidative stress, (ROS), and hypoxia lead to protein damage and aggregation, which induces autophagy. The substrate (cargo) for autophagy is degraded by lysosomal acid hydrolases, including cathepsins D, B, and L, after the fusion of lysosomes and autophagosomes to form autolysosomes. Rab7, LAMP-2A, and SNARE proteins are critical for the lysosome and autophagosome fusion process. Ubiquitin (Ub), LC3II, and p62 are complexed to the cargo and connect autophagy to the proteasomal clearance system. Macroautophagy is prevented in AMD because lysosomal lipofuscin disturbs cathepsin activity and autophagy flux. Fusion mechanisms in the RPE cells are under investigation. From Blasiak et al. [132], with the permission of the authors.

Otherwise, starvation and hypoxia, which can result from poor perfusion, are generally associated with increased amounts of ROS, which promote autophagy via several complex signaling mechanisms [138] (Figure 3). In response to OS, autophagy is significantly increased in an attempt to remove oxidatively damaged organelles such as mitochondria. At this time, accumulating evidence linking the impairment of autophagy with a range of age-related neurodegenerative diseases, including AMD, has suggested that autophagy occurs in the RPE to maintain homeostasis because these cells are exposed to sustained OS. However, insufficient digestion due to impaired autophagy or lysosomal degradation in the RPE can lead to an accumulation of damaged organelles, toxic proteins (including lipofuscin), and extracellular drusen deposits, all of which can contribute to RPE dysfunction or RPE cell death, which have been associated with the pathogenesis of AMD [139]. In addition, the AOXs of the retina (e.g., via macular molecules such as lutein and zeaxanthin) are reduced in AMD [140].

### 5.1. VEGF and AMD

VEGF is the most potent inducer of endothelial activation and angiogenesis. It is mainly expressed in retinal neurons and glial cells and is present in only scant amounts in blood vessels [141]. Under ischemic conditions, retinal expression and production of VEGF is increased [142]. This factor has also been implicated in the development of retinal neovascularization in ischemic retinopathies such as AMD [98]. Through a paracrine mechanism, VEGF binds to its cell-surface receptors, including VEGFR1/Flt-1, VEGFR2/Flk-1/KDR, and VEGFR3, and promotes endothelial cell survival, proliferation, migration, and tubular structure formation [142]. Among these receptors, VEGFR2 is the crucial receptor that mediates angiogenic and vascular permeability, whereas VEGFR3 mainly mediates lymphangiogenic functions. The activation of VEGFR1 plays a dual role and can either stimulate or inhibit angiogenesis, whereas the activation of VEGFR2 seems to only stimulate angiogenesis [143, 144].

Upon binding VEGF, VEGFR2 undergoes dimerization and autophosphorylation, resulting in the activation of its downstream kinases, including mitogen-activated protein kinase (MAPK), ERK1/2, p38, JNK, and PI3K/Akt, and of endothelial NO synthase (e-NOS), which may lead to further alterations in endothelial cell survival, proliferation, and migration. Recent reviews provide further information on this important factor [145, 146].

A constant oxygen supply is clearly essential for proper homeostasis and normal functioning in all retinal tissues. Cellular responses to reduced oxygen levels are mediated by the transcriptional regulator hypoxia-inducible factor-1 (HIF-1), a heterodimeric protein complex that consists of an oxygen-dependent subunit (HIF-1*α*) and a constitutively expressed nuclear subunit (HIF-1*β*). Under normoxic conditions, de novo synthesized cytoplasmic HIF-1*α* is degraded by the 26S proteasome. Under hypoxic conditions, HIF-1*α* is stabilized, binds to HIF-1*β*, and activates the transcription of various target genes. These genes play a key role in the regulation of angiogenesis in various visual pathologies, such as AMD [147].

### 5.2. AMD and the Complement Pathway

Growing evidence indicates that AMD is downstream of a chronic inflammatory condition in which activation of the immune system plays an important role. Metabolic products accumulate in the extracellular space between Bruch's membrane and the RPE and activate the complement system through a significant increase in OS, similar to the processes that occur in atherosclerosis or Alzheimer's disease [148]. These findings as well as those of many studies over the past decade have changed the understanding of the molecular mechanisms underlying AMD and led scientists to explore the targeting of specific molecular components of the complement pathway [149–153]. The complement system is a major component of innate immunity that plays a role in defense against invading microorganisms, the clearance of apoptotic cells, and the modulation of immune responses [154]. The complement cascade's four activation pathways converge upon a common terminal pathway that culminates in the formation of the cytolytic membrane attack complex (MAC). Binding of circulating C1q to antigen antibody complexes activates the* classical* pathway. The* lectin* pathway is activated by mannan-binding lectin following its recognition of, and binding to, molecular patterns on pathogen surfaces. The recently characterized* intrinsic* pathway is activated by proteases that cleave C3 and C5 directly. In contrast to the other three pathways, the* alternative* pathway is continuously active at a low level and is characterized by the spontaneous hydrolysis of C3 into the C3a and C3b fragments. C3b binds complement factor B (CFB), and once bound, CFB is cleaved by complement factor D (CFD) into Ba and Bb, thereby forming the active C3 convertase (C3bBb). C3bBb cleaves additional C3 molecules, which generates more C3a and C3b and thereby promotes further amplification of the cascade. In addition to the 30 or more complement components and fragments, numerous soluble and membrane-bound regulatory proteins modulate the complement system [153]. Complement factor H (CFH) is an important regulatory complement protein and is a major inhibitor of the alternative complement cascade that prevents excessive activation of the complement components. CFH regulates complement activity by inhibiting the activation of C3 to C3a and C3b and by inactivating existing C3b [154].

The discovery that drusen contain alternative complement pathway proteins led to the hypothesis that drusen could be involved in local complement-mediated inflammation [153]. The reports of an association between AMD and genetic variants in the* cfh* gene, a major inhibitor of the alternative pathway, support the inflammation model [154]. Other AMD risk variants have been found in genes underlying the alternative pathway, principally the formation of unstable C3 convertase, C3bBb, which cleaves C3 to generate the active segment C3b. Deposition of C3b on the target surface triggers the effector molecules C3a and C5a and the MAC, resulting in inflammation and cell lysis. In addition to CFH, several other AMD risk variants have been identified in genes underlying the alternative pathway. Variations in the factor B (BF) and complement component 2 (C2) genes are also associated with AMD [155].

A third line of evidence in support of complement involvement in AMD was provided by studies that showed that AMD patients have higher levels of complement activation products in their blood [156].

In 2001, data collected from the Age-Related Eye Disease Study (AREDS) revealed that patients who were treated with zinc, either alone or in combination with vitamins, displayed reduced progression to advanced AMD. AREDS results led to the recommendation that persons who are older than 55 years of age and who are at risk of developing advanced AMD should consider taking vitamin supplements plus zinc. A previous report published by the Blue Mountains Eye Study, a population-based study, confirmed the beneficial effect of zinc in AMD patients [157]. A recent study also provided evidence that daily administration of 50 mg of zinc sulfate can inhibit complement involvement in AMD patients who have increased complement activation [158].

### 5.3. AMD, RPE, and Daily and Circadian Rhythms

The retinal circadian system involves a unique structure. It contains a complete circadian system with multiple generation sites of numerous circadian rhythms, each of which deserves its own review. However, in the vertebrate retina, the intimate reciprocal relationship that exists between the neural retina and the underlying RPE is crucial for vision, while the diurnal and circadian rhythmicity of the RPE is critical for photoreceptor support and retinal function. Thus, in this section, we briefly review some of the rhythmic functions of the RPE that contribute to normal and pathological vision.

The association between AMD and biological rhythms has been poorly studied; however, there is a strong link between ocular physiology and circadian rhythms in both humans and animals. The renewal and elimination of aged photoreceptor outer segment tips by cells from the RPE is a daily rhythmic process that is crucial for long-term vision. Photoreceptors indefinitely renew their light-sensitive outer segments by disk shedding and the subsequent formation of new disks from the cilium of the inner segment. In higher vertebrates, outer segment renewal is synchronized by circadian rhythms [121, 159].

This shedding occurs once per day. In mice and rats, rod shedding is synchronized with light onset [160, 161]. To maintain the constant length of photoreceptors, the outer segment needs to be shed, and the formation of new outer segments must be coordinated.

The task of the adjacent RPE is to absorb shed POSs by phagocytosis and to recycle or digest their components. Outer segment renewal is crucial for photoreceptor function and survival, and a lack of efficient phagocytosis is sufficient to cause rapid photoreceptor degeneration by disk shedding [121, 162]. Photoreceptor disk shedding and subsequent phagocytosis by the RPE must be precisely regulated [163]. Alterations, such as delayed termination of shedding or defective digestion in the RPE, can cause the accumulation of lipofuscin [123]. Outer segment renewal and RPE phagocytosis are synchronized under circadian control and are triggered by the dark/light periods of the daily rhythm [163, 164].

Studies in higher vertebrates have revealed differences between cone- and rod-dominant species. Rod shedding mainly occurs in the morning, resulting in complementary RPE phagocytic activity by an increased number of phagosomes within the first 2 h after light onset, whereas cone shedding is more variable and mainly occurs either during the night or during the first 2 h after light onset [163, 164]. Any disruption in this process causes photoreceptor dysfunction and blindness in animal models and retinal disease in humans [93]. The synchronization of shedding with light seems to be crucial to photoreceptor physiology and survival because the accumulation of undigested material is detrimental to the RPE and retina and may contribute to the development or progression of AMD [123, 124]. These rhythms that synchronize with light are perceived by photosensitive retinal ganglion cells that contain the pigment melanopsin. Daily information is transmitted to the master circadian oscillator located in the SCN via the retinohypothalamic tract [66]. The action spectrum of light information for the circadian biological rhythm shows a peak at a shorter wavelength (464 nm) than that for visual information (approximately 555 nm).

Pivotal studies [161–164] have conclusively demonstrated that, in the rat retina, the diurnal rhythm of rod POS shedding and RPE phagocytosis is under circadian regulation. The mammalian retina displays persistent rhythmic activity even when it is isolated from the brain. In rats with transected nerves, POS shedding and RPE phagocytosis continue diurnally despite the loss of synaptic connections between the eye and the brain, suggesting that this rhythm is generated and controlled locally in the eye [165]. However, this rhythm cannot be reset by light unless the optic nerve remains intact [166]. Thus, the circadian renewal of POS involves both local control within the retina and central regulation by the brain.

The cellular and molecular mechanisms that underlie circadian regulation of shedding/phagocytosis are complex and are not the subject of this review. Numerous genes, proteins and signaling pathways play important roles in the engulfment of POS and the subsequent lysosomal degradation of spent photoreceptor disks within the RPE.

The lack of POS phagocytosis or digestion leads to photoreceptor dystrophy and blindness [122] due to the absence of the engulfment activity of the RPE. In rats that are deficient in MerTK, this absence causes dramatic and early onset retinal degeneration [122, 167]. Because the ingestion rate of POS by RPE cells exhibits a pronounced circadian rhythm that peaks around subjective dawn in both rat and mouse strains [159, 168], Prasad and colleagues [169] suggested that a feature of the TAM receptor system, such as ligand and/or receptor expression levels, might be regulated as a function of position in the circadian cycle.

Despite our vast knowledge of circadian biology and angiogenesis, the role of the circadian clock in the regulation of angiogenesis and vascular patterning remains poorly understood. Experimental animal models may help define the relationships between circadian rhythms and some retinal pathologies, such as AMD. Jensen et al. [170] showed that disruption of the circadian clock by both constant exposure to light and genetic manipulation of key genes in zebrafish led to impaired developmental angiogenesis. The disruption of crucial circadian regulatory genes, including* Bmal1* and* Period2*, resulted in either marked impairment or enhancement of vascular development. At the molecular level, these authors showed that the circadian regulator Bmal1 directly targets the promoter region of the* vegf* gene in zebrafish, leading to elevated VEGF expression. Interestingly, deletion of these E-boxes in the promoter region of the zebrafish* vegf* gene resulted in inactivation of the promoter. These findings can be reasonably extended to developmental angiogenesis in mammals and even to pathological angiogenesis in humans [171]. Disruption of the circadian clock system not only affects the physiological activity of an organism but also often leads to the onset, development and progression of various diseases [172].

An important circadian hormone that is involved in vertebrate retinal circadian rhythms is melatonin, which is synthesized and produced by photoreceptors and shows a clear daily rhythm, with an acrophase at night [173]. This indolamine seems to have protective effects on other retinal cell types, including RPE cells and photoreceptors. Melatonin protects cultured RPE cells from OS and ischemia-induced cell death [174, 175]. Several studies have reported that melatonin is involved in the pathogenesis of AMD. In 2005, Yi et al. [176] reported that daily administration of melatonin (3 mg) may protect the retina and delay the progression of AMD. Rosen et al. [177] reported that the production of melatonin is decreased in AMD patients compared with age-matched controls, suggesting that a deficiency in melatonin may play a role in the occurrence of AMD. A further indication of the possible role of melatonin in age-related pathologies is the observation that retinal melatonin synthesis decreases during aging. In 2012, Tosini et al. [178] proposed that melatonin could affect the circadian clocks in photoreceptors and RPE cells and could thereby affect metabolism in these cells.

Melatonin and dopamine, two regulatory signals that play important roles in retinal physiology, have been proposed to be involved in the control of circadian POS shedding and RPE phagocytosis. In the retina, the production and release of melatonin and dopamine are under circadian control [179, 180]. Although there is insufficient clinical and experimental evidence to demonstrate a direct relationship between melatonin, circadian rhythms, and AMD, some reports have suggested that the melatonin rhythm is reversed in AMD patients [181].

## 6. Conclusion

This review highlights the role of OS as one of the main causes of AMD etiologies. In this retinal disease, as in other metabolic and degenerative pathologies, complex interactions among metabolic, functional, genetic, and environmental factors create a platform for the development of chronic changes in the ocular structures of the macular region in addition to a strong age dependence. In addition to genetic predispositions, at least four processes contribute to the disease: lipofuscinogenesis, drusogenesis, local inflammation and neovascularization (in the case of the wet form), and immunological mechanisms.

The current pathophysiological conception of AMD assigns a primary role to the age-related, cumulative oxidative damage to the RPE that occurs due to an imbalance between the generation and elimination of ROS. In particular, lipofuscin has been hypothesized to be the primary source of ROS and to be responsible for both the cellular and extracellular matrixes alterations in AMD. However, there may also be an association between the increasing levels of environmental sun radiation, especially short wavelengths in the violet and blue spectrum, due to both the ozone hole and climate change over the last decades, particularly given that AMD remains the leading cause of irreversible vision loss among the elderly in developed nations. In 2004, the overall global prevalence of AMD was approximately 8.7 percent, and the number of AMD patients was projected to rise to 196 million people worldwide by 2020 and to 288 million by 2040 [182]. Interestingly, the association between AMD and circadian rhythms, particularly different RPE rhythms, suggests a role for the circadian clock in AMD-related circadian abnormalities, which have generally been considered to be a consequence of neurodegeneration. However, recent evidence suggests that circadian disruption might actually contribute to the degenerative process and thus might be a modifiable cause of cell or neural injury.

Circadian clock genes have been shown to regulate VEGF signaling in tumorigenesis [183], and dopamine has been shown to modulate the effects of VEGF receptor activation on vascular endothelial cells [184, 185]. Recent results also indicate that* Period* genes may play a similar role in regulating vascularization signals in the retina in retinopathy disease models [186]. Regardlessly, retinal clock gene expression is disrupted in proliferative neovascularizing diseases [187].

Although circadian disturbances due to aging and neurodegenerative diseases have been duly noted, a key question is whether these disturbances influence the pathology of AMD. This question deserves further investigation.

## List of Abbreviations

AMD: Age-related macular degeneration
AOX: Antioxidant defense
BRB: Blood-retinal barrier
CFH: Complement factor H
ETC: Electron transport chain
FR: Free radical
GPx: Glutathione peroxidase
HIF-1: Hypoxia-inducible factor-1
Melatonin: *N*-acetyl-5-methoxytryptamine
MAC: Membrane attack complex
mitoROS: Mitochondrially generated ROS
mtDNA: Mitochondrial DNA
NOX: NADPH oxidases
OS: Oxidative stress
PEDF: Pigment epithelium-derived factor
PMF: Proton motive force
POSs: Photoreceptor outer segment fragments
PUFAs: Polyunsaturated fatty acids
RET: Reverse electron transfer
RNS: Reactive nitrogen species
ROS: Reactive oxygen species
RPE: Retinal pigment epithelium
SCN: Suprachiasmatic nucleus
SODs: Superoxide dismutases
TTL: Transcriptional-translational feedback loop
VEGF: Vascular endothelial growth factor.
